# Performance and preference of problem-based learning (PBL) and lecture-based classes among medical students of Nepal

**DOI:** 10.12688/f1000research.107103.3

**Published:** 2023-10-04

**Authors:** Saroj Adhikari Yadav, Sangeeta Poudel, Oshna Pandey, Dhiraj Prasad Jaiswal, Bhupendra Prakash Malla, Brajesh Kumar Thakur, Swotantra Gautam, Sirish Raj Pandey

**Affiliations:** 1 Patan Academy of Health Sciences, Kathmandu, Nepal; 2 B P Koirala Institute of Health Sciences, Dharan, Nepal; 3 Nepal Cancer Hospital and Research Center, Kathmandu, Nepal

**Keywords:** medical education, undergraduate medical education, medical school, lecture-based classes, problem-based learning, PBL

## Abstract

**Background**: PBL (problem based learning) is new active learning educational strategy that has been extensively tested and used in recent years. Patan Academy of Health Sciences (PAHS) is one of medical schools from Nepal, a Low Income Country (LIC) implementing PBL for undergraduate medical education. This study aims to compare PAHS students' understanding and knowledge retention when taught through PBL and lecture-based classes and compare students' perceptions of PBL and lectures in medical education.

**Methods**: This is a cross-sectional study of medical students of a PBL based medical school in Nepal, a non-Western low-income country. Ethical approval was given by the institutional research committee of the Patan Academy of Health Sciences. Understanding and knowledge retention was assessed with 50 vignette-based multiple-choice questions, half of which were taught through PBL sessions, and the remaining half were taught in didactic lectures during basic science years of medical school. A separate pre-validated perception questionnaire was used to assess students' preferences regarding PBL and lectures.

**Results**: Out of 107 students, 99 participated in the understanding and knowledge retention questionnaires and 107 completed perception questionnaires. Understanding and knowledge retention of students was found to be the same for topics taught by PBL and lectures, with median scores of 17 and s16, respectively. PBL were mostly preferred for the physiology (59.81%), pathology (51.40%) and pharmacology (53.27%) concepts, and lectures were mostly preferred for the anatomy (78.50%), biochemistry (45.79%), and microbiology (42.99%) topics. Students wanted the same concepts to be taught through both PBL and lectures, especially for anatomy.

**Conclusions**: Understanding and knowledge retention is the same for topics taught by either PBL or lectures during the basic science years of undergraduate medical education. Students prefer PBL for physiology, pathology, and pharmacology-related concepts, conventional didactic lecture for physiology and microbiology, and a combination of lecture and PBL sessions for anatomy.

## Introduction

Sir William Osler, referred to as the father of modern medicine, emphasized teachers’ role in helping students to observe and reason. He recommended for teachers to abolish the traditional lecture method of instruction. ^1^ PBL (problem-based learning) is a newer active learning educational strategy that has been extensively tested and used in recent years. ^2^ PBL is an integral part of teaching in the undergraduate medical education of several medical schools around the globe, including the Patan Academy of Health Sciences (PAHS), a government medical school of a non-Western LIC (low-income country), Nepal. ^2^ The PBL process was pioneered by Barrows and Tamblyn at the medical school of McMaster University in Canada in the 1960s. ^3^ Students like PBL because it is student-focused, allows active learning, and leads to better understanding and retention of knowledge. ^4^^,^^5^

Students take more interest and responsibility for learning, look for resources like research articles, journals, internet, textbooks, etc. and themselves resolve the contextual problems given in PBL. ^6^ PBL enhances content knowledge and simultaneously fosters the development of communication, collaboration, problem-solving, critical thinking, and self-directed learning. ^7^^,^^8^ PBL emphasizes lifelong learning by developing the potential to determine goals, locate appropriate resources, and assume responsibility for what one needs to know. ^9^ It helps students for long-term knowledge retention and improves competency as physicians after graduation. ^10^^,^^11^ PBL has gradually been adopted by several medical schools around the globe for undergraduate education. ^12^^–^^14^

PBL encourages students to ask questions, search references, and think of logical answers. On the other hand, PBL is resource-intensive and requires more physical space, computer resources, and more staff to facilitate PBL sessions. ^15^ Students also report uncertainty, information overload, and inability to determine the required depth and relevance of information available. ^15^ This study aims to compare students’ understanding and knowledge retention of topics taught through PBL and lectures, and also compare students’ perception of PBL and traditional lectures, among the first two batches of medical students of PAHS who passed in 2016 and 2017. The data was collected before they graduated from PAHS.

## Methods

### Study design and participants

This study is a cross-sectional study performed in Nepal, a non-Western low-income country, among medical students of PAHS, where the hybrid PBL method is used during basic science years. All of the selected two batches of PAHS medical students were included in this study with their written informed consent. Students who did not give consent, students among the researcher team of this study, and students who participated in the pilot survey of questionnaires developed for this research were excluded from the study. The developed questionnaires were administered to 15 students who were randomly selected in the pilot study to establish the validity and feasibility. They were asked in detail about the questionnaire and any suggestions for revisions or editing needed. The pilot survey did not undergo any statistical comparisons. Only a few grammatical corrections were made after review and feedback from the pilot study. Subsequently, the final study was conducted on March 2014. Ethical approval was given by the Institutional Research Committee (IRC) of PAHS (IRC-PAHS) and research was carried out per relevant guidelines and regulations. ^16^

### Tools: validation, implementation, and analysis

Multiple-choice questions (MCQ) were used to assess understanding and knowledge retention, whereas a separate questionnaire was used to assess the preference for PBL and lectures. ^25^ The MCQ questionnaire for the assessment of understanding and knowledge retention had 50 vignette-based MCQs, half of which were from topics taught through PBL, and the remaining half were from topics taught through lectures. These MCQs were developed and validated by the students with the help of research advisors. The MCQ scores were converted into percentages and interpreted in terms of percentage: <60% = very low, 60-70% = low, 70-80% = moderate, 80-90% = high and 90-100% = very high. The perception questionnaire was compiled and discussed in the student research group and reviewed by the research advisors to establish content validity. It was administered to the 15 students to establish the face validity and feasibility. The perception questionnaire had 30 questions to be answered on a forced Likert scale, ranging from one (strongly agree) to four (strongly disagree). Students were allowed to explain or give opinions qualitatively in some questions of the perception questionnaire. Data entry and editing were done in an MS Excel spreadsheet and analyzed in the SPSS 13.0 software for Windows. Descriptive statistics (mean and percentage) and inferential statistics were used to compare perceptions. A p-value less than 0.05 was taken as a statistically significant result.

## Results

### Basic characteristics and information

Out of 107 students, 99 completed the understanding and knowledge retention questionnaires. ^25^ The mean age of the participants was 22 years. In total, 59.6% of respondents were male and 40 (40.4%) were female. The majority (67/99, i.e. 67.7%) of respondents have completed their schooling at a private school and 32 (32.3%) completed their schooling at a public school. About half (49.5%) were living in urban areas, while 24 (24.2%) and 26 (26.3%) were from semi-urban and rural areas, respectively. The majority (91, i.e. 91.9%) of the respondents were from a 10 + 2 high school science background, while only 8 (8.1%) were from 10 + 3 health sciences background.

### Understanding and knowledge retention of students

The normality test showed that the marks obtained by participants on topics taught via PBL and topics taught via lecture were not normally distributed (Shapiro-Wilk p-value = 0.015 for lectures and 0.024 for PBL). Thus, the median score was computed, which was 16 for lectures and 17 for PBL, as shown in Table 1.

**Table 1.** Multiple choice question (MCQ) median score by two teaching methods at Patan Academy of Health Sciences, Nepal.

	Lecture	Problem-based learning (PBL)
Total MCQs	25	25
Median score	16	17
Mann-Whitney U test	-0.706	
p-value	0.48	

Mann-Whitney U test was not statistically significant (p-value: 0.48), there was no difference in the median marks obtained in PBL and lecture methods.

### Perception of students

The perception scale was internally consistent as the coefficient alpha of the perception questionnaire was 0.893. The perception questionnaire answered on a forced Likert scale, ranging from one (strongly agree) to four (strongly disagree) showed that students mostly preferred physiology, pathology, and pharmacology-related concepts through PBL, whereas they preferred anatomy, biochemistry, and microbiology-related topics through lectures. Some students wanted to be taught via both PBL and lectures, especially for anatomy subjects ( Table 2).

**Table 2.** Subject preference to be taught using problem-based learning (PBL) and lectures (N = 107).

Subject	Preference for PBL (%)	Preference for lectures (%)	Preference for both (%)	No response (%)
Physiology	64 (59.81%)	50 (46.73%)	7 (6.54%)	0
Pathology	55 (51.40%)	53 (49.53%)	1 (0.93%)	0
Pharmacology	57 (53.27%)	48 (44.86%)	2 (1.87%)	4 (3.74%)
Anatomy	61 (57.01%)	84 (78.50%)	38 (35.51%)	0
Biochemistry	43 (40.19%)	49 (45.79%)	5 (4.67%)	20 (18.69%)
Microbiology	30 (28.04%)	46 (42.99%)	2 (1.87%)	33 (30.84%)
Introduction to Clinical Medicine (ICM)	29 (27.10%)	40 (37.38%)	2 (1.87%)	40 (37.38%)
Community Health Sciences (CHS)	21 (19.63%)	35 (32.71%)	1 (0.93%)	52 (48.60%)

Note: For multiple response items, respondents were allowed to select PBL or lecture or both.

The perception questionnaire also showed that neither PBL nor lectures were preferred for Community Health Science (CHS) and Introduction to Clinical Medicine (ICM), where many opted not to respond. Regarding CHS, students mentioned they learn public/community health better in community postings and lecture sessions with a group of faculty members as a part of Community-Based Learning Education (CBLE). Regarding ICM they prefer it on hospital wards and bedside teaching.

## Discussion

This study showed that students mostly liked being taught by both PBL and lectures. PBL was preferred for physiology, pathology, and pharmacology-related concepts, lectures were preferred for biochemistry and microbiology-related topics, and a combination of both for anatomy. Overall, the respondents wanted to be taught the same concepts via both PBL and lectures for anatomy. A meta-analysis by Nandi *et al.* compared the newer PBL curriculum and the conventional lecture-based mode of teaching undergraduate medical students. They concluded that a combination of both the conventional lecture-based and more recent PBL curricula would provide the most effective training for undergraduate medical students. ^17^ However, their findings were not subject-specific.

This study showed understanding and knowledge retention of students remained the same for topics taught by PBL compared to topics taught by lecture. There was no statistical difference in the median score obtained for PBL and lectures for the understanding and knowledge retention questionnaire (17 and 16, respectively). However, most other studies show better understanding and knowledge retention with PBL than with lectures. A study by Albanese *et al.* showed that the PBL students score higher than the students in traditional courses. They also concluded that the reason for higher scores in PBL are the learning competencies, problem-solving, self-assessment techniques, data gathering, behavioral science, etc. of PBL students. ^18^ Similarly, a study from Pakistan showed the mean score in the group exposed to PBL was 3.2 ± 0.8, while those attending lecture-based classes was 2.7 ± 0.8 (p = 0.0001). ^19^ Another study on mathematics students from Slovenia found that students exposed to PBL were better at solving more complex problems. ^20^

This study involved students taught through PBL in the first and second year of medical school i.e. basic science years, and showed equivalent results compared to lectures. However, another study on PAHS students showed that PBL imparts long-term knowledge retention through students’ active participation. ^21^ Wun *et al*. have also found that PBL started in the initial years of medical school is associated with more active participation, interaction, and collaboration among students, and PBL students score higher too. ^22^ Another study on nursing students found that all students with higher or lower grades showed a significant increase in scores among students in the PBL group. Still, only students with higher grades showed a notable increase in scores among students in the lecture group. Learning motivation was also significantly higher in the PBL group (t = 2.608, p = 0.012). ^23^

A few of the respondents qualitatively reported in this study that some students in the PBL group worked harder than other members of the same group to prepare and participate in discussions. They also found the time allocated for each topic was not sufficient at times. Silva *et al.* reported that teamwork and the time involved can limit PBL learning. ^24^ According to Wood, major disadvantages to this process involve the tutor facilitation and utilization of excessive resources. ^15^

## Conclusion

Students’ understanding and knowledge retention is the same for topics taught by PBL compared to topics taught by conventional didactic lecture. PBL is preferred for physiology, pathology, and pharmacology-related concepts. In contrast, traditional lectures were preferred for biochemistry and microbiology-related topics, and a combination of lecture and PBL sessions were preferred for anatomy during the basic science years of undergraduate medical education. Students prefer community-based programs and lecture sessions delivered by a group of faculty members for CHS. In contrast, they prefer a bedside teaching and hospital ward-based teaching methodology for ICM rather than lectures and PBL.

## Data availability

### Underlying data

Figshare: Raw data and Questionnaire of research “Performance and Preference of Problem-Based Learning (PBL) and Lecture-Based Classes Among Medical Students of Nepal.” https://doi.org/10.6084/m9.figshare.17286902.v1. ^23^

This project contains the following underlying data:

• Raw Data of PBL Research.xls
• Descriptive Analysis of PBL Research.doc

### Extended data

Figshare: Raw data and Questionnaire of research “Performance and Preference of Problem-Based Learning (PBL) and Lecture-Based Classes Among Medical Students of Nepal.” https://doi.org/10.6084/m9.figshare.17286902.v1. ^23^

This project contains the following extended data:

• Preference questionaire of PBL Research.doc
• Understanding questionnaire by MCQ of PBL Research.docx

Data are available under the terms of the Creative Commons Attribution 4.0 International license (CC-BY 4.0).
