# Supplementary material for: Performance and preference of problem-based learning (PBL) and lecture-based classes among medical students of Nepal
Source: F1000Res. 2023 Oct 4;11:183. Originally published 2022 Feb 14. [Version 3] doi: 10.12688/f1000research.107103.3 (PMC10955184; doi:10.12688/f1000research.107103.3)
Supplement: Peer review history

# Reviewer report: Reviewer response for version 3

**DOI:** 10.5256/f1000research.156930.r255160

**Published:** 2024-03-20

**Authors:** Ashlesha Chaudhary

**Affiliations:** 1 Nepal Medical College and Teaching Hospital,, Kathmandu, Nepal

The article is well written. The methodology is sound, and the results are clearly presented. The statistical analysis appears appropriate and detailed. The authors have addressed key aspects of student preferences and learning outcomes, providing valuable insights for curriculum development and teaching practices. Overall, I recommend accepting this article for publication.

Is the work clearly and accurately presented and does it cite the current literature?

Yes

If applicable, is the statistical analysis and its interpretation appropriate?

Yes

Are all the source data underlying the results available to ensure full reproducibility?

Yes

Is the study design appropriate and is the work technically sound?

Yes

Are the conclusions drawn adequately supported by the results?

Yes

Are sufficient details of methods and analysis provided to allow replication by others?

Yes

Reviewer Expertise:

Medical Education, Psychiatry

I confirm that I have read this submission and believe that I have an appropriate level of expertise to confirm that it is of an acceptable scientific standard.

# Reviewer report: Reviewer response for version 3

**DOI:** 10.5256/f1000research.156930.r212106

**Published:** 2023-10-25

**Authors:** Mamata Chimmalgi

**Affiliations:** 1 Department of Anatomy, Sree Gokulam Medical College and Research Foundation, Trivandrum, Kerala, India

I have seen the remarks of the authors for the queries. They are all satisfactory and hence the same can be accepted for indexing with no further suggestions from me. Best wishes for the authors.

Is the work clearly and accurately presented and does it cite the current literature?

Yes

If applicable, is the statistical analysis and its interpretation appropriate?

Yes

Are all the source data underlying the results available to ensure full reproducibility?

Yes

Is the study design appropriate and is the work technically sound?

Yes

Are the conclusions drawn adequately supported by the results?

Yes

Are sufficient details of methods and analysis provided to allow replication by others?

Yes

Reviewer Expertise:

NA

I confirm that I have read this submission and believe that I have an appropriate level of expertise to confirm that it is of an acceptable scientific standard.

# Reviewer report: Reviewer response for version 2

**DOI:** 10.5256/f1000research.133438.r136042

**Published:** 2022-04-29

**Authors:** Babu Raja Maharjan

**Affiliations:** 1 Department of Biochemistry, School of Medicine, Patan Academy of Health Sciences, Lalitpur, Nepal

Some revision have been made in the manuscript following my previous comments. However following two comments have not been addressed.

 1. When was MCQs and questionnaire administered for this study?

 The outcome of MCQs score will depend on whether they are administered immediately or a long time after the completion of basic science. In the discussion itself authors have mentioned the dichotomous result on knowledge retention from the literature. Stating administered time of MCQs will also will help authors to justify their indifference in knowledge retention between PBL and lecture.

 2. What about Likert scale questionnaire result?

 In the preference questionnaire, there are Likert scale questionnaire for PBL and lecture. But its result is not mentioned. Why?

Is the work clearly and accurately presented and does it cite the current literature?

Yes

If applicable, is the statistical analysis and its interpretation appropriate?

Yes

Are all the source data underlying the results available to ensure full reproducibility?

Yes

Is the study design appropriate and is the work technically sound?

Partly

Are the conclusions drawn adequately supported by the results?

Partly

Are sufficient details of methods and analysis provided to allow replication by others?

Yes

Reviewer Expertise:

NA

I confirm that I have read this submission and believe that I have an appropriate level of expertise to confirm that it is of an acceptable scientific standard, however I have significant reservations, as outlined above.

# Response

**Published:** 2023-10-02

**Authors:** Saroj Adhikari Yadav

**Affiliations:** Patan Academy of Health Sciences School of Medicine, Nepal

Dear Dr. Maharjan,

 Thank you for your meticulous review of the paper and for providing feedback for significant improvement.

 Regarding question number 1, we appreciate your observation. The data was collected in May 2014, and we have now included this information in the manuscript to ensure completeness and transparency.

 Regarding question number 2, we would like to clarify that the Likert scale questionnaire data was indeed analyzed and the results were incorporated into the Results section of the manuscript. As mentioned in the manuscript, our findings indicated that students mostly preferred PBL for physiology, pathology, and pharmacology-related concepts, while they preferred lectures for biochemistry and microbiology-related topics. Additionally, a combination of both PBL and lectures was preferred for anatomy. Furthermore, we observed that neither PBL nor lectures were strongly preferred for Community Health Science (CHS) and Introduction to Clinical Medicine (ICM) topics.

 We apologize if the presentation of this information in the manuscript was not as clear as expected. To address this, we have reviewed the manuscript, and rewrote some parts "Perception of students" topic in the results section of the manuscript ensuring that the Likert scale questionnaire results are appropriately emphasized in the revised version to provide a more detailed and comprehensive understanding of students' preferences for different subjects and teaching methods.

 Thank you for your valuable feedback.

 Yours Sincerely,

 -Dr. Adhikari Yadav.

# Reviewer report: Reviewer response for version 1

**DOI:** 10.5256/f1000research.118167.r134523

**Published:** 2022-04-20

**Authors:** Babu Raja Maharjan

**Affiliations:** 1 Department of Biochemistry, School of Medicine, Patan Academy of Health Sciences, Lalitpur, Nepal

This paper follows the IMRAD guidelines. Abstract and introduction is well written. But the limitations of the study need to be stated and following comments need to be addressed before indexing.

 Perception questionnaire was mentioned in the research method, but its finding was not described in the result section. Please provide a reason for this inconsistency.

 It is not stated when the questionnaire and MCQ test was administered. Recall bias will be there if it is administered a long time after the completion of basic science years where PBL is being implemented. This in turn will affect the measurement of performance by MCQ score and preference of PBL/Lecture for various subjects. Therefore, it should be clearly mentioned as the limitation of the study and current finding is based on students preference and performance of PBL measured after ?? time period.

 Furthermore, the drawing of comparative inference on the preference of subjects for either PBL or Lecture is inappropriate. Questionnaire that was used in the study independently asked the preference of subjects for PBL and Lecture to the participants hence inference cannot be drawn if a certain subject has been preferred more or less for PBL over lecture. With the given study design, valid inference that can be drawn will be just the preference of various subjects to PBL and the preference of different subjects to Lecture but not comparison between PBL and lecture for a subject as mentioned in discussion and conclusion.

 In discussion section, all of a sudden there is presentation of qualitative findings while it was neither mentioned in method section or result section. This needs to be consistent.

Is the work clearly and accurately presented and does it cite the current literature?

Yes

If applicable, is the statistical analysis and its interpretation appropriate?

Yes

Are all the source data underlying the results available to ensure full reproducibility?

Yes

Is the study design appropriate and is the work technically sound?

Partly

Are the conclusions drawn adequately supported by the results?

Partly

Are sufficient details of methods and analysis provided to allow replication by others?

Yes

Reviewer Expertise:

Health Professions Education, Obesity, Adipocyte Biology, Insulin resistance, Diabetes

I confirm that I have read this submission and believe that I have an appropriate level of expertise to confirm that it is of an acceptable scientific standard, however I have significant reservations, as outlined above.

# Response

**Published:** 2022-04-22

**Authors:** Saroj Adhikari Yadav

**Affiliations:** Patan Academy of Health Sciences School of Medicine, Nepal

Dear Dr. Maharjan,

 Thank you for your comments and suggestion.

 Regarding your comments and reservation-

 1. I am including your feedback and uploading a new version of the manuscript.

 2. Preference data is included in the 3rd and 4th paragraphs of the results.

 3. Data were collected before the 1st and 2nd batch graduated in 2016 and 2017. So, I assume, recall bias should not be that significant. I am including this in the updated version of the manuscript.

 4. We have mentioned in the 2nd paragraph of the method section that "Students were allowed to explain or give opinions qualitatively in the perception questionnaire."

 Thank you once again.

# Reviewer report: Reviewer response for version 1

**DOI:** 10.5256/f1000research.118167.r126411

**Published:** 2022-04-01

**Authors:** Mamata Chimmalgi

**Affiliations:** 1 Department of Anatomy, Sree Gokulam Medical College and Research Foundation, Trivandrum, Kerala, India

Saroj Adhikari Yadav et al. in their article ‘Performance and preference of problem-based learning (PBL) and lecture-based classes among medical students of Nepal’ have compared the learning outcome following PBL and didactic lecture and assessed preference of the students regarding the two methods. The article is written adhering to IMRAD structure and elaborates the findings of a cross sectional study conducted in PAHS in Nepal.

- Abstract is structured and draws attention to the aims of the study and the correctly drawn conclusions. 
- Introduction segment enumerates the merits and challenges of PBL and proceeds to list the aims of this study in that context. 
- Methods segment elaborates on inclusion and exclusion criteria; developing MCQ questionnaire and perception questionnaire; establishing validation of the questionnaire; implementation and analysis. However, time intervals between the T/L methods and the administration of the questionnaire may be added to understand whether immediate learning or long-term retention were being studied - although the discussion segment mentions another study at PAHS addressing the long term retention, the method followed in the current study is missing.
- In the Results segment, inference drawn regarding the performance may be included under ‘understanding and knowledge retention of students’ before proceeding to the results - e.g. ‘learning outcome following PBL was similar to lecture’ or ‘students performance following PBL was comparable to performance following didactic lecture’….…
- Regarding perception of students - both in results and in discussion segments, other parameters assessed by the perception questionnaire - unless deliberately omitted - may be included.
- Conclusion segment effectively and correctly sums up the findings.
- Under References, is there a need to add the last reference by the authors (no. 25), when the same are shared under data availability?

Overall, a well-written, clear and concise article.

Is the work clearly and accurately presented and does it cite the current literature?

Yes

If applicable, is the statistical analysis and its interpretation appropriate?

Yes

Are all the source data underlying the results available to ensure full reproducibility?

Yes

Is the study design appropriate and is the work technically sound?

Yes

Are the conclusions drawn adequately supported by the results?

Yes

Are sufficient details of methods and analysis provided to allow replication by others?

Yes

Reviewer Expertise:

Anatomy, Histology and Medical education

I confirm that I have read this submission and believe that I have an appropriate level of expertise to confirm that it is of an acceptable scientific standard, however I have significant reservations, as outlined above.

# Response

**Published:** 2022-04-22

**Authors:** Saroj Adhikari Yadav

**Affiliations:** Patan Academy of Health Sciences School of Medicine, Nepal

Dear Dr. Chimmalgi,

 Thank you for your comments and suggestion. I am including your feedback and uploading a new version of the manuscript.

 Thank you once again.
